# Fatty Acid Binding Protein 1 Is Related with Development of Aspirin-Exacerbated Respiratory Disease

**DOI:** 10.1371/journal.pone.0022711

**Published:** 2011-08-04

**Authors:** Tae-Hoon Kim, Ji-Yeon Lee, Jong-Sook Park, Sung-Woo Park, An-Soo Jang, Jae-Yong Lee, Jang-Yul Byun, Soo-Taek Uh, Eun-Suk Koh, Il Yup Chung, Choon-Sik Park

**Affiliations:** 1 Genome Research Center for Allergy and Respiratory Disease, Soonchunhyang University Bucheon Hospital, 1174, Jung-Dong, Wonmi-Gu, Bucheon, Gyeonggi-Do, South Korea; 2 Department of Otorhinolaryngology-Head and Neck Surgery, Soonchunhyang University Bucheon Hospital, 1174, Jung-Dong, Wonmi-Gu, Bucheon, Gyeonggi-Do, South Korea; 3 Division of Allergy and Respiratory Medicine, Soonchunhyang University Hospital, 657, Hannam-Dong, Yongsan-Gu, Seoul, South Korea; 4 Department of Pathology, Soonchunhyang University Bucheon Hospital, 1174, Jung-Dong, Wonmi-Gu, Bucheon, Gyeonggi-Do, South Korea; 5 Division of Molecular and Life Sciences, College of Science and Technology, Hanyang University, 1271 Sa-1-dong, Ansan, Gyeonggi-Do, South Korea; Oregon Health and Science University, United States of America

## Abstract

**Background:**

Aspirin-exacerbated respiratory disease (AERD) refers to the development of bronchoconstriction in asthmatics following the ingestion of aspirin. Although alterations in eicosanoid metabolites play a role in AERD, other immune or inflammatory mechanisms may be involved. We aimed to identify proteins that were differentially expressed in nasal polyps between patients with AERD and aspirin-tolerant asthma (ATA).

**Methodology/Principal Findings:**

Two-dimensional electrophoresis was adopted for differential display proteomics. Proteins were identified by liquid chromatography-tandem mass spectrometry (LC-MS). Western blotting and immunohistochemical staining were performed to compare the amount of fatty acid-binding protein 1 (FABP1) in the nasal polyps of patients with AERD and ATA. Fifteen proteins were significantly up- (seven spots) or down-regulated in the nasal polyps of patients with AERD (n = 5) compared to those with ATA (n = 8). LC-MS revealed an increase in seven proteins expression and a decrease in eight proteins expression in patients with AERD compared to those with ATA (P = 0.003–0.045). FABP1-expression based on immunoblotting and immunohistochemical analysis was significantly higher in the nasal polyps of patients with AERD compared to that in patients with ATA. FABP1 was observed in epithelial, eosinophils, macrophages, and the smooth-muscle cells of blood vessels in the polyps.

**Conclusions/Significance:**

Our results indicate that alterations in 15 proteins, including FABP1, may be related to the development of AERD.

## Introduction

Aspirin-exacerbated respiratory disease (AERD) refers to the development of bronchoconstriction in asthmatics individuals following the ingestion of aspirin (acetylsalicylic acid) or other non-steroidal anti-inflammatory drugs. This syndrome is characterized by the “aspirin triad,” which consists of aspirin hypersensitivity, bronchial asthma, nasal polyposis, and chronic hyperplastic eosinophilic sinusitis [1], [2]. As is true for other asthmatic individuals, the airways of patients with AERD show signs of persistent inflammation with marked eosinophilia, epithelial disruption, cytokine production, and the up-regulation of inflammatory molecules. The over- or under-production of critical mediators, including leukotrienes, lipoxins, thromboxane, and prostaglandins, in the metabolism of arachidonic acid probably accounts for this susceptibility to aspirin. Variation within the genes of the arachidonate pathway is responsible for changes in the production and metabolism of these mediators. Polymorphisms in such genes as *LTC4S* [3], *ALOX5* [4], *PTGER2* [5], *PTGER3* [6], *TBXA2R* [7] and *CYSLTR2* [8] have been revealed to be associated with AERD.

ASA and sodium salicylate have been reported to inhibit activation of the transcription factors NFKB [9]. ASA also regulate IL4 expression by altering the availability of transcription factors to the regulatory elements in the IL4 promoter [10] and inhibit IL4– and IL13–induced activation of STAT6 [11]. These observations suggest that aspirin regulates a more complex network of biochemical and cellular events than was initially thought. To characterize this network, high-throughput techniques, including proteomics and gene expression analysis, are needed to identify genes related to AERD. Three reports have utilized microarray technology to examine gene expression and methylation patterns in nasal polyp tissues from AERD [12], [13], [14]. These reports indicated that nasal polyps have characteristic transcriptional signatures compared with normal tissues; however, no global studies of protein expression in nasal polyps have been undertaken for AERD. In this study, we adopted a proteomics-based approach to investigate the pathobiology of and to develop a marker for aspirin hypersensitivity.

## Results

### 2-DE of nasal polyps and protein identification in ATA and AERD samples by LC-MS

**Figure 1:**
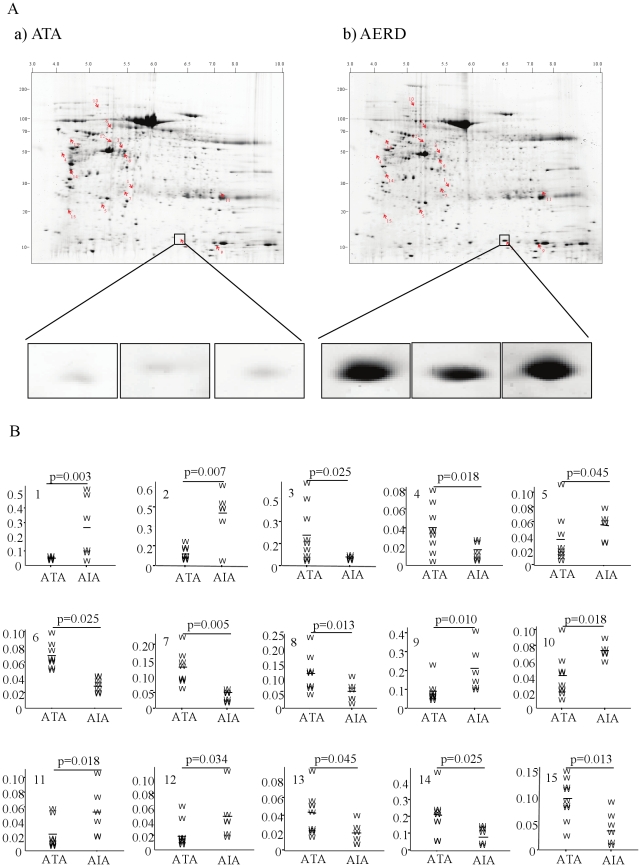
Photographs of two-dimensional electrophoresis (2-DE) separation of nasal polyps proteins obtained from ATA (n = 8) and subjects with AERD (n = 5). (A) one ATA, and one patient with AERD. Nasal polyp proteins (1 mg) were focused on a pH 3–10 immobilized pH gradient strip and then separated by 7.5–20% gradient SDS-PAGE and visualized as described in the Methods. Protein spots identified by LC-MS (arrows) are labeled with numbers. Spot #2 is enlarged in small boxes. (B) The Y-axis of each graph represents the relative intensity of the spot (%). P-values represent the difference between the ATA and AERD samples.

The nasal polyp samples were separated in the first dimension on IPG gel strips over a pH range of 3 to 10, followed by separation in the second dimension by 7.5–20% homogeneous SDS-PAGE (Figure 1). After Coomassie blue staining and image analysis, a mean number of 984 spots (range 853–994) in the ATA samples and 907 spots (range 790–984) in the AERD samples were detected in 1 mg of nasal polyp. The relative intensities were significantly higher in seven spots and lower in eight spots in the AERD samples compared to the ATA samples. These spots were excised from the gels and incubated with trypsin for in-gel digestion and then identified by LC-MS. All of the identified spots were localized between a pH of 4 and 8 with a molecular mass range of 10 to 150 kDa (Figure 1A). The relative intensities (%) of the spots are expressed as median values and interquartile ranges for each group (Figure 1B). Fifteen proteins showed significant differences in relative intensity between the two groups according to the Mann-Whitney U-test (p<0.05). The results of these analyses are summarized in Table 1. The relative intensity of spot #1 (chaperonin group 2) was ten times higher in the AERD patients than in the ATA patients (p = 0.003). FABP1, carbamyl phosphate synthetase I, aldehyde dehydrogenase 4A1 precursor, betaine-homocysteine methyltransferase isoform CRA b, immunoglobulin gamma light chain, and glyceraldehyde-3-phosphate dehydrogenase were more abundant in the AERD samples than in the ATA samples. In contrast, the relative intensities of the other eight spots were significantly lower in the AERD group than in the ATA group (Figure 1 and Table 1). They were identified as beta actin, transketolase, transketolase variant, heat shock 70 kDa protein 9 precursor, UDP-glucose pyrophosphorylase 2 isoform a, heat-responsive protein 12, KIAA1361 protein, and zinc finger protein 609 (Table 1).

**Table 1:** List of differentially expressed proteins identified by LC-MS.

*No.*	*Protein name*	*Accession No.*	*Determined sequence*	*MW(kDa)*	*Relative intensity*	*P-value*	*Function*
				*PI*	*ATA AERD*		
1	Chaperonin	31542947	R.IQEIIEQLDVTTSEYEK.E	27.9/5.7	0.022<0.223	0.003	*Protein folding*
2	FABP1	116284334	K.YQLQSQENFEAFMK.A	15.7/6.4	0.065<0.389	0.007	*Fatty acid meabolism*
3	Beta actin	4501885	K.DLYANTVLSGGTTMYPGIADR.M	50.1/5.4	0.191>0.036	0.025	*Cytoskeleton*
4	Transketolase	37267	K.SKDDQVTVIGAGVTLHEALAAAELLK.K	50.0/4.1	0.040>0.013	0.018	*glycolysis*
5	Carbamyl phosphate synthetase I	219553	K.GYSFGHPSSVAGEVVFNTGLGGYPEAITDPAYK.G	24.8/5.2	0.030<0.053	0.045	*Energy metabolism*
6	Transketolase variant	62898960	K.SKDDQVTVIGAGVTLHEALAAAELLK.K	50.0/5.4	0.063>0.043	0.025	*glycolysis*
7	Heat shock 70 kDa protein 9 precursor	24234688	K.VIAVYDLGGGTFDISILEIQK.G	27.3/5.4	0.138>0.053	0.005	*Anti-inflammatory*
8	UDP_glucose pyrophosphorylase 2 isoform a	48255966	K.TLDGGLNVIQLETAVGAAIK.S	80.7/5.3	0.120>0.053	0.013	*Glucose transfer*
9	Aldehyde dehydrogenase 4A1 precursor	25777734	K.ETLQLVDSTTSYGLTGAVFSQDKDVVQEATK.V	12.9/7.2	0.094<0.196	0.01	*Proline degradation*
10	Betaine-homocysteine methyltransferase, isoform CRA b	119616238	R.LNAGEIVIGDGGFVFALEK.R	121.04/5.1	0.043<0.074	0.018	*methyl transfer*
11	Ig γ light chain	34427	K.LLIYSNNQRPSGVPDR.F	28.8/7.3	0.023<0.053	0.018	*Inflammatory*
12	Glyceraldehydes-3-phosphate dehydrogenase	31645	K.WGDAGAEYVVESTGVFTTMEK.A	61.2/5.3	0.022<0.050	0.034	*Energy metabolism*
13	Heat-responsive protein 12	5032215	K.TTVLLADINDFNTVNEIYK.Q	63.3/4.4	0.042>0.021	0.045	*unknown*
14	KIAA1361 protein	7243103	K.FQQHIQAQQK.K	38.3/4.4	0.208>0.076	0.025	*microtubule affinity regulating kinase kinase*
15	Zinc finger protein 609	71725360	K.QKPSIPPTLTK.A	21.5/4.2	0.092>0.033	0.013	*unknown*

### Expression of FABP1 in nasal polyps from AERD and ATA patients

**Figure 2:**
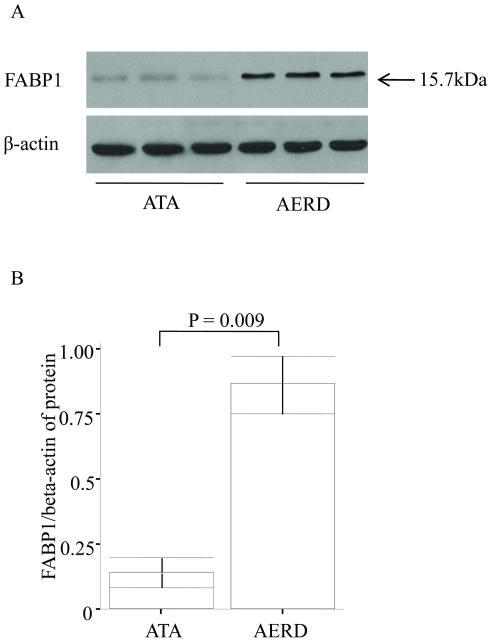
Comparison of FABP1 expression in ATA and AERD patients. FABP1 abundance was measured in nasal polyp extract lysates of ATA (n = 3) and AERD (n = 3) patients using a mouse anti-human FABP1 monoclonal antibody. (A) Representative figure and (B) bar graph of the Western blot analysis of plasma from AERD and ATA samples.

**Figure 3:**
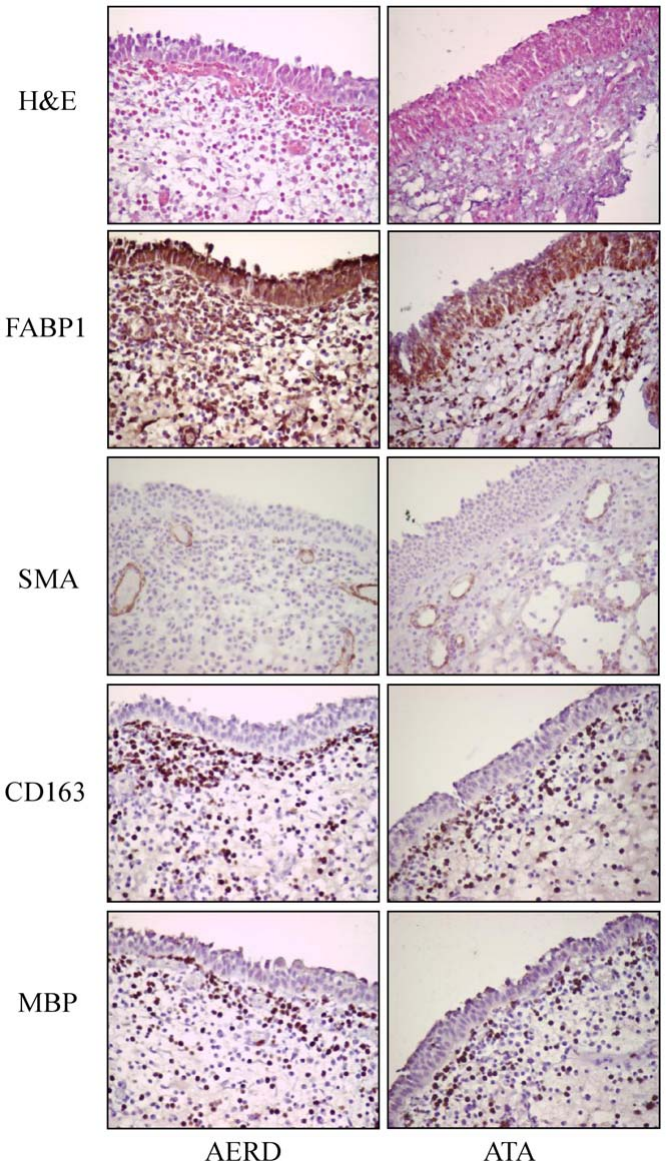
Representative figure showing the immunohistochemical analysis of nasal polyps from ATA (n = 3) and AERD (n = 3) patients. A mouse anti-human FABP1 monoclonal antibody (1∶100 dilution), goat anti-human CD163 polyclonal antibodies (1∶100 dilution), goat anti-human MBP polyclonal antibodies (1∶100 dilution), and a mouse anti-human alpha smooth muscle actin monoclonal antibody (1∶50, clone 1A4, DAKO, Glostrup, Denmark) were used. Magnification: 400×.

To investigate whether FABP1 expression was altered in nasal polyps, we performed Western blotting and immunohistochemical staining using nasal polyps obtained from similar subjects with ATA (n = 5) and AERD (n = 8). A 15.7 kDa band representing FABP1 was present by Western blotting in both groups (Figure 2A). The density normalized with beta actin was significantly higher in the nasal polyps from the AERD patients than in those from the ATA patients (0.84±0.09 vs. 0.13±0.03, respectively, p = 0.009; Figure 2B). Immunohistochemical staining of serial sections for FABP1, SMA, CD163, and MBP was performed on 4-µm sections of paraffinized nasal polyp samples. Immunohistochemical staining showed markedly increased expression of FABP1 in the nasal polyps of the AERD patients compared to those with ATA. FABP1 was strongly expressed in the epithelial cells, CD 163 positive macrophages, MBP positive eosinophils, and SMA positive smooth muscle cells of blood vessel (Figure 3).

### Plasma concentration of FABP1 in subjects with AERD or ATA

**Figure 4:**
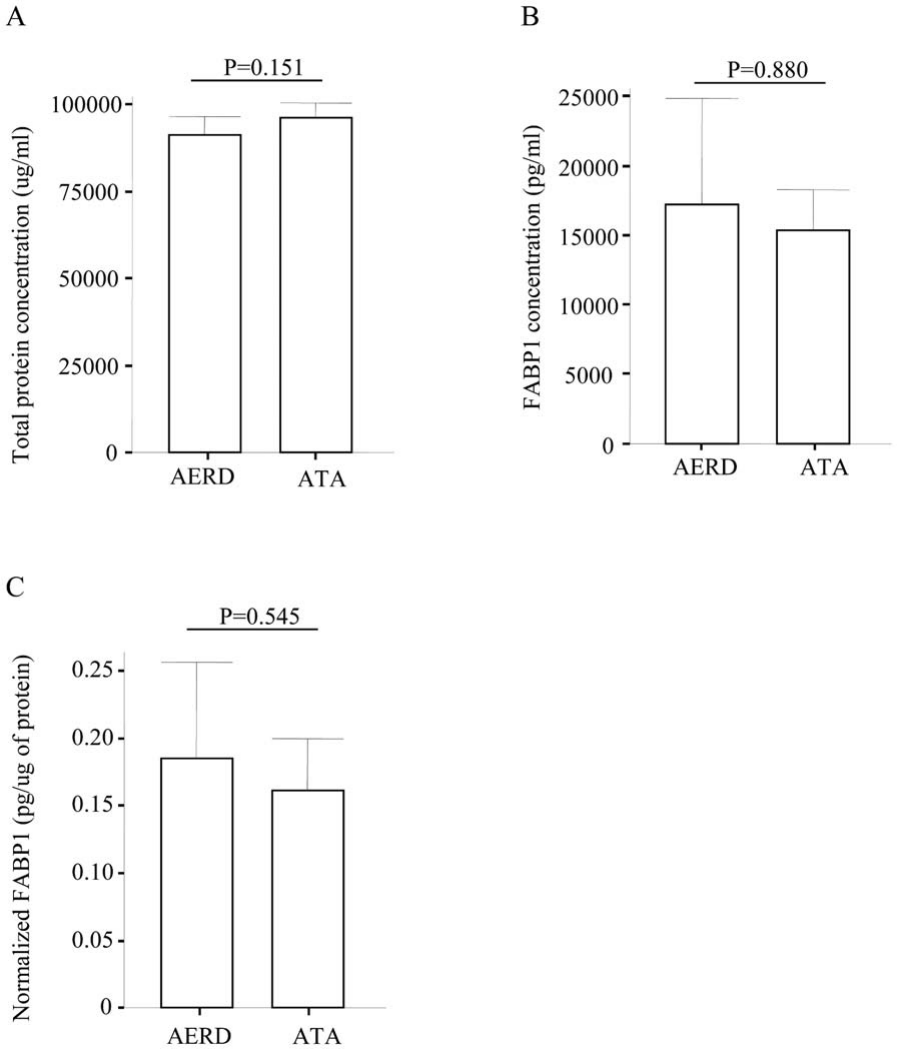
Comparison of FABP1 levels in plasma from AERD (n = 10) and ATA (n = 10) patients. (A) Total protein, (B) FABP1, and (C) normalized FABP1 concentrations with total plasma protein.

The plasma concentration of FABP1 in the AERD and ATA subjects was found to be similar by ELISA (17,245±2,607 pg/ml vs. 15,304±1,476 pg/ml, respectively, p = 0.880; Figure 4B). The normalized concentration of FABP1 (with total protein) was similar in both groups (0.18±0.04 pg/ug vs. 0.16±0.02 pg/ug, respectively, p = 0.545; Figure 4C).

## Discussion

In the present study, we use 2-D gels to distinguish AERD from ATA based on protein abundance. We identified 15 proteins that exhibited differential expression between ATA and AERD patients. In a previous study using plasma from patients with AERD or ATA, we found that the amounts of three proteins (complement C3 fragments, apolipoprotein, and modified albumin) differed between the two groups [15]. Although complement components are regarded as a candidate participant in the pathogenesis of AERD, additional searches for other molecular makers of AERD have been done using new biosources.

AERD is characterized by extensive mucosal inflammation in the lower (asthma) and upper (rhinitis and nasal polyps) airways [2]. The nasal polyps of AERD patients show similar pathological characteristics, including the infiltration of inflammatory cells into the bronchial mucosa [16]. The inflammatory cell infiltrate sets up an extensive network of cellular interactions, which are thought to promote nasal polyp proliferation. Thus, nasal polyps are a good biosource for the study of AERD. To identify new markers to better understand the molecular basis of AERD, we adopted a proteomics-based approach. This is the first report to show, in a single map, the different proteins expressed in nasal polyp tissues from patients with AERD or ATA.

We found 15 spots that were differentially expressed in AERD and ATA patients. LC-MS showed that, in contrast to the changes in complement proteins and apolipoproteins in the plasma, several other types of proteins were expressed differentially in the nasal polyps. The amount of FABP1 was six times higher in the AERD samples than in the ATA samples (p = 0.007) based on 2-DE, and eight times higher by Western blot analysis. Our immunohistochemical results indicate that FABP1 is expressed more abundantly in the nasal polyps of AERD patients. In particular, FABP1 strongly expresses in epithelial cells, macrophages, eosinophils, and blood vessel smooth muscle cells.

Fatty acid trafficking in cells is a complex and dynamic process that affects many aspects of cellular function. Linoleic and arachidonic acids can be metabolized to a diverse and large family of bio-active lipid mediators called eicosanoids, which may function as pro- and anti-inflammatory mediators [17]. FABPs are small, abundant cytoplasmic proteins that reversibly bind saturated and unsaturated long chain fatty acids, eicosanoids, and other lipids. FABPs play a role in trafficking of intracellular arachidonic acid metabolism and in the conversion of fatty acids to eicosanoid intermediates and in the stabilization of leukotrienes [18]. Among the mediators of the eicosanoid pathway, LTA4 is an intermediate in the formation of biologically active leukotrienes. LTA4 in the binding site of FABP is stabilized for subsequent biochemical processing to LTB4 and cysteinyl leukotrienes [18]. The striking stabilization of LTA4 by various FABP proteins suggests that this family plays a central and previously unrecognized role in leukotriene biosynthesis in the cell.

An additional function of FABP is the reduction of 15-lipoxygenase (LO) activity [19]. The end products of 15-LO are lipoxin and aspirin-triggered lipoxin, which have anti-inflammatory actions. Taken together, the elevation of FABP may switch the tissue environment to LTB4 or cysteinyl leukotriene over-production and down-regulation of the lipoxin family, which has been demonstrated in nasal polyps or airways of AERD patients. Of note, our immunohistochemical results demonstrate that FABP1 is significantly elevated in the nasal polyps of AERD patients compared to ATA patients. This finding highlights the need for a renewed focus on candidate modulators of inflammatory responses and arachidonic acid metabolism by FABP1.

Because L-FABP1 is produced mainly by the liver, it has been used as a marker of liver injury. Recently, it was shown that L-FABP1 levels were altered in patients with septic shock and in those with nephropathy [20]. Lipid peroxidation and lipid accumulation could be the primary cause of FABP1 excretion into urine, and urinary shedding of FABP1 may protect against tubular cell injury. This prompted us to measure FABP1 in the plasma of our subjects. The levels were similar in both types of patients. This indicates that plasma FABP1 may have no effect on the level in nasal polyps of the subjects in the present study.

The reason for the increase in FABP1 in the nasal polyps of the AERD patients is unknown. In addition, it is unknown whether the elevation in FABP1 is the cause of aspirin hypersensitivity or the result of extensive inflammation in the nasal polyps of patients with AERD. FABPs themselves are controlled by transcription factors, including the PPAR family [21]. Interestingly, aspirin up-regulates PPAR alpha/gamma in various cells via the over-production of eicosanoid metabolites, including 15-hydroxyeicosatetraenoic acid (15-HETE), and 15-deoxy-12, 14-prostaglandin J2 (15d-PG J2). Recently, a polymorphism in the gene encoding PPAR gamma was revealed to be associated with aspirin hypersensitivity [22]. Taken together, over-production of the PPAR family via enhanced 15-HETE production in AERD may induce FABP over-expression in the airways leading to exaggerated conversion to cysteinyl leukotrienes from stabilized LTA4-FABP complexes.

Other proteomic studies have been performed on the nasal polyps of patients with chronic sinusitis using 2-DE, and several proteins expressed differentially between chronic sinusitis and normal mucosa [23], [24] before and after steroid treatment have been identified [25]. Although 20 to 30 differentially expressed protein spots were detected, a change in FABP abundance was not demonstrated. The reason for this may be the fact that aspirin sensitivity is limited to about 30% of nasal polyposis cases [2].

Another interesting finding is the change in chaperones in the AERD samples compared to the ATA samples. The relative intensity of chaperonins following 2-DE was ten times higher in the AERD samples than in the ATA samples. Chaperonins are protein complexes that assist the folding of nascent, non-native polypeptides into their native and functional state. Molecular chaperones such as Hsp70 and TCP-1 ring complex (TRiC)/chaperonin containing TCP-1 (CCT) are implicated as potent modulators of misfolding diseases. These chaperones suppress the toxicity of disease proteins and modify early events in the aggregation process in a cooperative and sequential manner, reminiscent of their functions in *de novo* protein folding. The cytosolic stress pathway or heat shock response results in the nearly instantaneous induction of the expression of such chaperones as heat shock proteins [26]. In the present study, the levels of heat shock 70 kDa protein 9 precursor and heat-responsive protein 12 were two times higher in ATA than in AERD samples. The elevated cytoplasmic Hsp70 levels are thought to play a protective role against cell damage induced by exogenous stresses, including heat shock, tumor necrosis factor, oxidative stress, cytostatic drugs, and radiation. After treatment with a nontoxic dose of ASA, Hsp70 membrane expression was up-regulated, especially as membrane-bound Hsp70. Thus, the change in chaperonins in the present study may indicate the altered celluar response of nasal polyps to aspirin in AERD as compared to ATA. Alternatively, disease processes themselves might cause, or worsen, the chaperone deficiency. The DNA binding activity of the transcription factor HSF-1 decreases with age in rat hepatocytes, causing a continuous decline in the ability to induce the expression of genes encoding chaperones during the cytosolic stress response [26]. In the present study, the mean age was not different between the AERD and ATA patients, which can exclude the possibility of age effects on Hsp70 levels. Another interesting finding is the change in several intracellular enzymes, including carbamyl phosphate synthetase I, aldehyde dehydrogenase 4A1 precursor, betaine-homocysteine methyltransferase isoform CRA b, transketolase, UDP-glucose pyrophosphorylase 2 isoform a, and glyceraldehyde-3-phosphate dehydrogenase. All of the above proteins except transketolase and UDP-glucose pyrophosphorylase 2 isoform a were increased in AERD.

Nitric oxide (NO) is a well-known active factor in endothelial-derived relaxing factor. Carbamoyl phosphate synthetase 1 (CPS1) is an enzyme that catalyzes the production of carbamoyl phosphate, a precursor of arginine and NO [27]. Thus, the CPS1 affects the L-citrulline/L-arginine cycle and subsequent NO production. The amount of CPS1 was about 150% higher in the AERD samples than in the ATA samples in the present study. Interestingly, numerous dilated blood vessels were observed in the AERD samples compared to the ATA samples, which provides indirect evidence of a possible role for CPS1 in the nasal polyps of AERD patients. Aldehyde dehydrogenase 4A1 precursor, betaine-homocysteine methyltransferase isoform CRA b, transketolase, UDP-glucose pyrophosphorylase 2 isoform a, and glyceraldehyde-3-phosphate dehydrogenase were differentially expressed in the AERD and ATA patients. Aldehyde dehydrogenase 4A1 precursor is related to a mitochondrial matrix NAD-dependent dehydrogenase, which catalyzes the second step of the proline degradation pathway, converting pyrroline-5-carboxylate to glutamate [28]. Betaine-homocysteine methyltransferase 2 is a sulfur-containing amino acid that plays a crucial role in methylation reactions [29]. Transketolase is a thiamine-dependent enzyme that links the pentose phosphate pathway to the glycolytic pathway [30]. UDP-glucose pyrophosphorylase 2 isoform a plays an important intermediary role in mammalian carbohydrate interconversions [31]. These findings indicate the possibility of alterations in energy metabolism in the nasal polyps of AERD patients compared to ATA patients.

In summary, to identify nasal polyp proteins that were differentially expressed between AERD and ATA patients, we performed 2-DE with LC-MS. Seven proteins were significantly up-regulated while eight proteins were down-regulated in the nasal polyps of AERD patients (n = 5) compared to ATA patients (n = 8). Among them, FABP1 levels were significantly higher in the nasal polyps of the AERD patients compared to the ATA patients based on our Western blot results. Immunohistochemical staining showed strong staining in the nasal polyps of the AERD samples compared to the ATA samples. However, the plasma FABP1 levels were similar in both. This indicates that an increase in FABP1 abundance in nasal polyps may be related to the development of AERD and may be a useful marker for diagnostic and therapeutic trials.

## Materials and Methods

### Ethics statement

Consent to participate in the study was obtained in writing from all subjects. The protocols used in this study were approved by the Soonchunhyang Bucheon Hospital's ethics committee. Thirteen nasal polyp tissues were obtained from the biobank at the Genome Research Center for Allergy and Respiratory Diseases (Soonchunhyang Bucheon Hospital), South Korea.

### Subjects

All patients met the criteria for asthma definition by the Global Initiative for Asthma (GINA) guidelines [32]. All patients had a history of dyspnea and wheezing during the previous twelve months plus one of the following: 1)>12% increase plus 200 mL following inhalation from a short-acting bronchodilator, or 2)<10 mg/mL PC_20_ methacholine. Twenty-four common inhalant allergens (e.g., dust mites [*Dermatophagoides farinae* and *D. pteronyssinus*], *Aspergillus*, cat fur, dog fur, cockroaches, grasses, trees, and ragweed pollen) (Bencard Co. Ltd., Brentford, UK) were used for skin prick tests. Atopy was defined as having a wheal reaction either equal to or greater than histamine or 3 mm in diameter. Total IgE was measured using the CAP system (Pharmacia Diagnostics, Uppsala, Sweden). The asthmatics had experienced no exacerbation of their condition or a respiratory tract infection during the six weeks preceding the oral aspirin challenge (OAC). The reactions of the patients to the OAC were categorized as follows: ≥15% decrease in FEV_1_ or naso-ocular reactions (AERD) and <15% decrease in FEV_1_ without naso-ocular or cutaneous reactions (ATA). OAC was performed with increasing doses of aspirin using methods slightly modified from those described previously [10], [33], [34]. Briefly, patients with a history of aspirin hypersensitivity were given 30 mg orally. Respiratory and nasal symptoms, blood pressure, external signs (urticaria and angio-oedema) and FEV_1_ were documented every 30 min for a period of 1.5 h. In the absence of any symptom or sign suggesting an adverse reaction after 1.5 h, increasing dosages of aspirin (60 mg, 100 mg, 300 mg, and 400 mg) were administered and the same measurements were repeated every 1 h until the patient developed the reaction. Those having no history were started on 100 mg of aspirin and gradually increased the dosage of aspirin as 200 mg, 350 mg, and 450 mg until the patient developed the reaction. If no reaction occurred 4 h after the final dose, the test was deemed negative. Aspirin-induced bronchospasm, reflected by a decline (%) in FEV_1_, were calculated as the pre-challenge FEV_1_ minus the post-challenge FEV_1_ divided by the pre-challenge FEV_1_. OAC reactions were categorized into three groups as follows: (1) 15% or greater decrease in FEV_1_ or nasal reactions (aspirin-intolerant asthma [AERD]) or (2) less than 15% decrease in FEV_1_ without naso-ocular or cutaneous reactions (aspirin-tolerant asthma [ATA]). (3) less than 15% decrease in FEV_1_ with cutaneous reactions (aspirin-induced urticaria) [AIU]).

Peripheral venous blood was obtained before OAC. Peripheral venous blood was collected before aspirin challenge. Peripheral blood mononuclear cells were separated on Ficoll -Hypaque solution. All subjects gave informed consent to participate in the study. The protocols were approved by the local ethics committees of hospital. Diagnoses of nasal polyps and chronic rhinosinusitis were made based on the patients' symptoms as assessed from their documented medical histories, the presence of endoscopically visible nasal polyps arising from the middle nasal meatus, and involvement of the ethmoidal and maxillary sinuses as visualized by limited CT scanning of the paranasal sinuses. The clinical characteristics of the subjects are summarized in Table 2. All polyps were kept at −80°C until needed. Consent to participate in the study was obtained in writing from all subjects. The protocols used in this study were approved by the Hospital's ethics committee.

**Table 2:** Clinical characteristics of the study subjects.

	ATA	AERD
No	8	5
Age, yr (mean ± SE)	51.9±6.6	43.8±3.2
Sex (M/F)	4/4	2/3
Smoker (%)	25%	-
Height (cm)	164.2±3.0	163.6±1.9
Weight (kg)	63.6±3.4	63.4±4.5
FVC (%, predicted)	96.7±6.5	89.8±4.4
FEV1 (%, predicted)	104.0±9.4	87.6±6.4*
Positive skin test (%)†	50	60

Values are mean ± SE. P-values are obtained using the *t*-test or the x^2^-test between AERD and ATA.

ATA: aspirin tolerant asthma, AERD : aspirin-exacerbated respiratory disease,

*P<0.05 compared with ATA.

† D.f, D.p, Strawdust, Haydust, Cockroach mix, Chrysanthemum P, Aster P, Cat fur, Ragweed polle.

### Two-dimensional electrophoresis (2-DE), protein identification by nano liquid chromatography-tandem mass spectrometry (LC-MS), and a database search

2-DE was performed using Immobiline DryStrips (24 cm, pH 3–10, pH 3–7; Amersham Biosciences, Uppsala, Sweden) for isoelectric focusing (IEF). DryStrips were first rehydrated in 450 mL of rehydration buffer containing 8 M urea, 2% CHAPS, 13 mM DTT, 1.2% IPG buffer, and a trace of bromophenol blue then mixed with each sample. IEF was carried out for a total of 146 kVh using the IPGphore system (Amersham Biosciences) with 1 mg of nasal polyp. Following IEF, the gel strips were equilibrated twice for 10 min with gentle shaking in equilibration buffer containing 50 mM Tris-HCl buffer (pH 8.8), 6 M urea, 20% glycerol, 0.1% SDS, and 1% DTT. In the second equilibration buffer, DTT was replaced with 2.5% iodoacetamide to remove excess DTT. After equilibration, the proteins were separated on 5–18.5% SDS polyacrylamide gels using the Ettan Dalt II system (Amersham Pharmacia Biotech, Inc., Uppsala, Sweden). At the end of each run, the gel was stained with Coomassie brilliant blue G-250. Digitized images of the stained gels were analyzed using the analysis program imagemaster 2D (version 4.0; Amersham Pharmacia Biotech, Inc.). Coomassie-stained spots on the gels of thirteen subjects were quantified on the basis of the normalized volume (i.e., the spot volume divided by the total sum of all spot volumes.).

Differentially expressed protein spots were excised from the 2-D gels, cut into smaller pieces, and digested with trypsin (Promega) [15], [35]. LC-MS was performed using Agilent Nanoflow Proteomics Solution and the Agilent 1100 Series nano LC system for MS/MS coupled through an orthogonal nanospray ion source to an Agilent 1100 Series LC/MSD Trap XCT ion trap mass spectrometer. The nano LC system was operated in sample enrichment/desalting mode with a ZORBAX 300SB-C18 enrichment column (0.3×50 mm, 5 um). Chromatography was performed using a ZORBAX 300SB-C18 (75 um×150 mm) nanocolumn. The column was eluted with a gradient beginning with an isocratic application of 3% solvent B (0.1% formic acid in acetonitrile) and 97% solvent A (0.1% formic acid in water) for 5 min. The gradient mixture was then changed to 10% B over 5 min (from 5 to 10 min), to 45% B over 40 min (10 to 50 min), to 90% B (isocratic) for 5 min (55 to 60 min), and to 3% B over 1 min (60 to 61 min). Finally, the column was washed with 3% B for 10 min.

The LC/MSD Trap XCT was operated in the unique peptide scan auto-MS mode. The ionization mode was positive nanoelectrospray with an Agilent orthogonal source. The drying gas flowed at 5 L/min at a temperature of 300°C. Vcap was typically 1,800–1,900 V with skim 1 at 30 V; the capillary exit was offset at 75 V. The trap drive was set at 85 V with averages of one or two. Ion charge control was on with a maximum accumulation time of 150 ms; the smart target was 125,000 and the MS scan range was 300–2,200. Automatic MS/MS was performed in ultrascan mode with the number of parents two, averages of two, a fragmentation amplitude of 1.15 V, SmartFrag on (30–200%), active exclusion on (after two spectra for 1 min), prefer +2 on, MS/MS scan range of 100–1,800, and ultrascan on. Each acquired spectrum was searched against the non-redundant protein sequence database using Spectrum Mill software.

### Western blotting and immunohistochemical staining of nasal polyps for FABP1 expression

FABP1 expression was analyzed by Western blotting and immunohistochemical staining with a mouse anti-human FABP1 monoclonal antibody. The protein samples were fractionated by 15% SDS-PAGE and transferred to nitrocellulose membranes (Amersham Pharmacia Biotech, Inc.). The membranes were incubated in a blocking solution containing a 1∶500 dilution of mouse anti-human FABP1 monoclonal antibody (Abcam, Cambridge Science Park, Cambridge, UK) and then incubated with blocking solution containing a 1∶5,000 dilution of horseradish peroxidase-conjugated polyclonal anti-mouse IgG antibodies. Enhanced chemiluminescence (ECL) detection was performed according to the manufacturer's instructions (Boehringer Mannheim, Mannheim, Germany). For the immunohistochemical analysis of FABP1, nasal polyp tissue on the slides was treated with 0.3% H_2_O_2_ for 20 min to block endogenous peroxidase and then incubated at 4°C overnight with a mouse anti-human FABP1 monoclonal antibody (1∶100 dilution; Abcam), goat anti-human CD163 polyclonal antibodies (1∶100 dilution; Santa Cruz Biotechnology Inc., Santa Cruz, CA, USA), goat anti-human MBP polyclonal antibodies (1∶100 dilution; Santa Cruz Biotechnology Inc.), and a mouse anti-human alpha smooth muscle actin monoclonal antibody (1∶50, clone 1A4, DAKO). The slides were incubated with an ABC kit (Vector Laboratories, Burlingame, CA, USA), and the color developed with 3,3′-diaminobenzidine tetrachloride (Zymed Laboratory Inc., South San Francisco, CA, USA). Other immunohistochemical analyses included the use of a mouse anti-human alpha smooth muscle actin monoclonal antibody (1∶50, clone 1A4, DAKO). For signal detection, the sections were incubated with the DAKO Real EnVision Detection System (K5007) according to the manufacturer's protocol. Staining for the expression of CD163, MBP, and alpha smooth muscle actin was performed for macrophages, eosinophils, and smooth muscle or myofibroblasts, respectively.

### Measurement of plasma FABP1 by ELISA

Plasma FABP1 was measured using a quantitative sandwich enzyme immunoassay kit according to the manufacturer's instructions (HyCult Biotechnology, Uden, The Netherlands). The lower limit of detection for FABP1 was 102 pg/mL. Values below this limit were assumed to be zero for statistical analysis. The inter- and intra-assay coefficients of variance were below 15%.

### Statistical analysis

Statistical analysis was performed with SPSS 8.0. For the analysis of spot intensity on the 2-D gels and determination of the FABP1 concentration, the Mann-Whitney test (two-sample rank sum test) was used to analyze differences between the two groups (AERD vs. ATA). All data are expressed as the mean ± SE; significance was defined as p<0.05.
